# Next-Generation Sequencing on Circulating Tumor DNA in Advanced Solid Cancer: Swiss Army Knife for the Molecular Tumor Board? A Review of the Literature Focused on FDA Approved Test

**DOI:** 10.3390/cells11121901

**Published:** 2022-06-11

**Authors:** Damien Vasseur, Hela Sassi, Arnaud Bayle, Marco Tagliamento, Benjamin Besse, Christophe Marzac, Ahmadreza Arbab, Nathalie Auger, Sophie Cotteret, Mihaela Aldea, Félix Blanc-Durand, Arthur Géraud, Anas Gazzah, Yohann Loriot, Antoine Hollebecque, Patricia Martín-Romano, Maud Ngo-Camus, Claudio Nicotra, Santiago Ponce, Madona Sakkal, Olivier Caron, Cristina Smolenschi, Jean-Baptiste Micol, Antoine Italiano, Etienne Rouleau, Ludovic Lacroix

**Affiliations:** 1 Medical Biology and Pathology Department, Gustave Roussy, F-94805 Villejuif, France; hela.sassi@gustaveroussy.fr (H.S.); christophe.marzac@gustaveroussy.fr (C.M.); ahmadreza.arbab@gustaveroussy.fr (A.A.); nathalie.auger@gustaveroussy.fr (N.A.); sophie.cotteret@gustaveroussy.fr (S.C.); etienne.rouleau@gustaveroussy.fr (E.R.); ludovic.lacroix@gustaveroussy.fr (L.L.); 2 AMMICa UAR3655/US23, Gustave Roussy, F-94805 Villejuif, France; 3 Drug Development Department (DITEP), Gustave Roussy, F-94805 Villejuif, France; arnaud.bayle@gustaveroussy.fr (A.B.); arthur.geraud@gustaveroussy.fr (A.G.); patricia.martin-romano@gustaveroussy.fr (P.M.-R.); maud.ngocamus@gustaveroussy.fr (M.N.-C.); claudio.nicotra@gustaveroussy.fr (C.N.); santiago.ponce@gustaveroussy.fr (S.P.); antoine.italiano@gustaveroussy.fr (A.I.); 4 Oncostat U1018, Inserm, Université Paris-Saclay, Équipe Labellisée Ligue Contre le Cancer, F-94805 Villejuif, France; 5 Medical Oncology Department, Gustave Roussy, F-94805 Villejuif, France; marco.tagliamento@gustaveroussy.fr (M.T.); benjamin.besse@gustaveroussy.fr (B.B.); mihaela.aldea@gustaveroussy.fr (M.A.); anas.gazzah@gustaveroussy.fr (A.G.); yohann.loriot@gustaveroussy.fr (Y.L.); antoine.hollebecque@gustaveroussy.fr (A.H.); olivier.caron@gustaveroussy.fr (O.C.); 6 Gynecological Cancer Unit, Department of Medicine, Gustave Roussy, F-94805 Villejuif, France; felix.blanc-durand@gustaveroussy.fr; 7 Dermatology Unit, Oncology Department, Gustave Roussy, F-94805 Villejuif, France; madona.sakkal@gustaveroussy.fr (M.S.); cristina.smolenschi@gustaveroussy.fr (C.S.); 8 Department of Hematology, Gustave Roussy, F-94805 Villejuif, France; jeanbaptiste.micol@gustaveroussy.fr

**Keywords:** cfDNA, liquid biopsy, next-generation sequencing, molecular tumor board, FDA-approved

## Abstract

FDA-approved next-generation sequencing assays based on cell-free DNA offers new opportunities in a molecular-tumor-board context thanks to the noninvasiveness of liquid biopsy, the diversity of analyzed parameters and the short turnaround time. It gives the opportunity to study the heterogeneity of the tumor, to elucidate complex resistance mechanisms and to adapt treatment strategies. However, lowering the limit of detection and increasing the panels’ size raise new questions in terms of detection of incidental germline alterations, occult malignancies and clonal hematopoiesis of indeterminate potential mutations. In this review, after a technological discussion and description of the common problematics encountered, we establish recommendations in properly using these FDA-approved tests in a molecular-tumor-board context.

## 1. Introduction

Since the development of large next-generation sequencing (NGS) panels adapted for the analysis of cell-free circulating DNA (cfDNA), liquid biopsy represents an attractive alternative to tissue biopsy in terms of reduced invasiveness, number of targeted genes, sensitivity and diversity of analyzed parameters (single-nucleotide variants (SNV), fusions, copy number variations (CNV), tumor mutational burden (TMB), microsatellite status). Even if tissue molecular profiling is still the gold standard, a major advantage of liquid biopsy is to reveal the heterogeneity of the tumor, as it offers a complete overview of the primary tumor and its associated metastases. Moreover, it may overcome sampling limitations experienced with tissue biopsy in cases where tissue biopsy is not feasible without risk for the patient [1] or has failed [2], or when the quality and quantity of the DNA (e.g., insufficient tumor cellularity or old archival samples) hamper sequencing [3,4,5].

Several putative applications have been identified for use in context of diagnostics [6], prediction of patient’s prognosis or response to treatments [7], monitoring of treatment [8] and resistance detection [9], while also tracking tumor evolution [10].

For these reasons, since the approval of cfDNA-based comprehensive genomic profiling by the Food and Drug Administration (FDA), this type of assay has been increasingly used for molecular screening and results are usually discussed in molecular tumor boards (MTB). The patient selection criteria that will be discussed during the MTB vary greatly from one center to another. In our experience, only patients with advanced solid tumors are discussed. The most important indication is the identification of actionable molecular targets or molecular resistance mechanisms, offering treatment alternatives as well as possibilities for inclusion in clinical trials. Currently, many clinical trials integrate cfDNA analysis. For instance, in the phase III SOLAR-1 trial, which evaluated alpelisib plus fulvestrant versus fulvestrant in *PIK3CA*-mutated, hormone-receptor-positive advanced breast cancer, *PIK3CA* mutations detected in plasma were probably correlated with greater disease burden and better response to the combination of alpelisib and fulvestrant [11]. Other screening studies such as the observational basket trial for advanced solid malignancies GOZILA were based on cfDNA, resulting in shortened screening duration and improved trial enrollment rate compared to tissue profiling [12]. Going further it is also worth noting the efforts of projects such as the Circulating Cell-free Genome Atlas study aiming at early detection of cancer using deep cfDNA sequencing [13].

However, the extension of the number of screened genes, as well as the very low limit of detection (LoD) of these NGS based cfDNA assays, i.e., the lowest allelic frequency of the target alteration that can be detected with a 95% detection rate, lead to new molecular information, such as mutations related to clonal hematopoiesis (CH) or incidental discovery of germline alterations that could complexify the interpretation for clinical decision. In this review we focus on the two FDA tests approved to date, discuss the added value of these large cfDNA assays and the questions raised by unexpected additional information in the context of MTB.

## 2. Parameters Analyzed in FDA-Approved Pan-Cancer Panels

Many pan-cancer commercial liquid biopsy panels are available, but until now only Guardant360^®^ CDx (Guardant Health, Inc.; Redwood, CA, USA) (G360) [14,15] and FoundationOne^®^ Liquid CDx (Foundation Medicine, Inc.; Cambridge, MA, USA) (F1LCDx) [16] obtained FDA approval. No specific approvals have currently been validated in Europe, and these assays tend to be performed for patient care within the context of clinical trial screening. These tests require an optimal volume of whole blood varying from 17 to 20 mL, and both allow the detection of SNV, CNV, fusions and microsatellite instability in a short turnaround time (from 7 to 14 days) (Table 1).

In a pan-cancer study performed on patients aiming to detect kinase fusion on plasma samples using F1LCDx, cfDNA was detected in 88% of plasma samples (/samples) and at least one genomic alteration was identified in 82.2% of the samples (/) for an average of 3.3 alterations per case [17].

### 2.1. Tumor Fraction

The tumor fraction is an estimation of tumoral cfDNA. This is provided in the clinical report interpretation only by F1LCDx. The tumor fraction is estimated based on a normalized coverage level across the genome, which gives a representation of tumor aneuploidy.

If the proportion of tumoral cfDNA is low, the tumor fraction is estimated based on the allelic frequencies of the somatic mutations excluding common and rare germline variants [17]. Low tumor fraction is a main cause of sample failure and impacts the concordance between tissue and liquid biopsy for SNV, TMB, microsatellite instability, CNV and fusion. In the absence of any relevant tumor variant, the question of the presence of tumor DNA in the extracted cfDNA remains open, and the analysis of another sample is often requested. Conversely, an elevated tumor fraction reflects a high cfDNA content and increases confidence in the identified alterations.

### 2.2. Tumor Mutation Burden

Neither tissue nor blood TMB (bTMB) calculation and interpretation have been standardized, despite many proposition and harmonization projects [18,19,20].

Currently, the only FDA test evaluating bTMB is the F1LCDx test. The blood bTMB is calculated taking into account all the mutations (synonymous or not) identified with an allelic frequency higher than 0.5% and not reported as a potential germline polymorphism in dbSNP and ExAC databases or as a classical oncogenic driver. The number of mutations identified with these filters is divided by the number of bases sequenced: 0.75 Mb. Then, the results are expressed as the number of mutations per Mb [16].

With the very low LoD of these cfDNA genomic profiling, there is a major risk of overestimating the bTMB value. Indeed, bTMB can be up to 2.4 times higher than tissue TMB with a positive linear correlation between the two values (r^2^ = 0.62). Interestingly, in the same study, patients treated with immunotherapy showing discordant TMB values—high bTMB and low tissue TMB—had longer time to treatment failure than the concordant patients with high bTMB and high tissue TMB (227 vs. 183 days) [21]. In another study, an increased progression-free survival benefit with atezolizumab was observed in lung cancer, establishing a cut-off at 16 mutations/Mb for bTMB. A positive correlation between blood and tissue TMB was reported (spearman rank correlation = 0.64) [22]. The cut-off is 10 mutations/Mb for tissue TMB in the same context [23].

### 2.3. Microsatellite Instability Detection (MSI)

Microsatellite status is analyzed by both FDA-approved assays. The F1LCDx assay focuses on approximately 2000 repetitive loci containing at least 5 repeat units of mono, di or tri nucleotides. If more than 0.5% of the studied loci are unstable, the sample is considered MSI-High [16]. For G360, MSI status is determined focusing on 99 microsatellite loci containing short tandem repeats of length 7 or more [24].

Pan-cancer concordance between G360 and tissue MSI determination has been estimated at 87% (71/82, 95% CI, 77–93%) for MSI-high patients and 99.5% (863/867, 95% CI, 98.7–99.8%) for MSS patients, with an overall accuracy of 98.4% (934/949, 95% CI, 97.3–99.1%). Comparing in detail cfDNA MSI evaluation with different tissue techniques, concordance of the status was 83% with immunohistochemistry (IHC) (93/112), 97.4% with polymerase chain reaction (PCR) test (450/462) and 98% with NGS (239/244). However, even if in most cases, concordance seems robust; the results are more likely to be discordant in cases with low tumor fraction (less than 0.2%) [24].

### 2.4. Single-Nucleotide Variation

**Figure 1:**
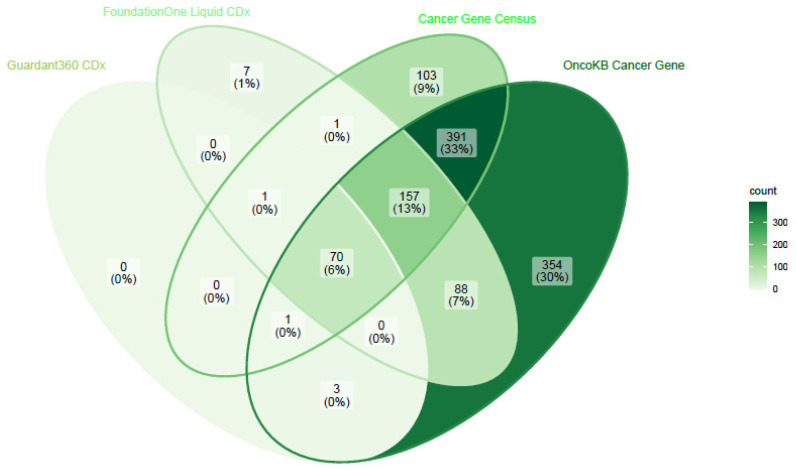
Venn diagram representing overlap of genes studied for SNV in two FDA-approved panels and two commonly used reference gene lists.

The two FDA-approved panels differ in the number of genes studied (311 genes for F1LCDx vs 73 for G360) (Figure 1). Nevertheless, the 71 genes in common are currently sufficient to cover the major actionable targets used for solid tumor analysis [25].

Both companies use molecular barcodes in order to detect mutations at very low frequencies. A recent study compared cfDNA NGS performed with or without molecular barcodes on 12 patients suffering from NSCLC or biliary-pancreatic cancer. In this small cohort, 7 mutations were identified when NGS was performed without molecular barcode and 17 when the technology was used. The use of molecular barcode ensures a better sensitivity [26]. Using a similar technology, F1LCDx is able to detect a mutation in 95% of cases if the VAF (variant allele frequency) is over a frequency ranging from 0.40 to 0.82%. The performances are almost similar for G360 with a probability of 95% to detect mutations with a VAF ranging from 0.20 to 0.25%.

Concordance between mutations identified in blood and in matching tumor tissue are widely documented. In colorectal cancer, concordance of *KRAS* mutations vary between 86.4% and 92% [27], estrogen receptor 1 (*ESR1*) mutation in metastatic breast cancer is concordant in 74.3% of patients [28] and actionable *EGFR* in NSCLC was reported to be concordant in 79% of patients [29]. However, some recent results focused on 100 patients with lung adenocarcinomas (71 samples collected at diagnosis and 29 after progression) showed poorer performance. Only 47.4% of the 78 mutations were identified by both tissue and plasma NGS, 5.1% were detected only by plasma NGS and 47.4% by tissue NGS only. The accuracy of the NGS performed on tissue was 96% with a sensitivity of 94.9%, whereas the accuracy of plasmatic NGS was 63% with a sensitivity of 52.6% [30].

These results must be balanced and always interpreted taking into account the tumor fraction. In a recent study, the overall concordance for TP53 alterations was 59.5% (47/79 mutations) when cfDNA fraction was <1.5% and reached 86.3% (69/80 mutations) when tumor fraction was ≥1.5% (*p*-value < 0.001) [31].

The lowering of LoD for the cfDNA-based NGS assays allows the detection of mutations down to 0.1% VAF, but not for all SNV or SNV types. This is why some authors pointed out the limits of concordance between the different tests. A comparison between G360 and another CLIA-certified test on 40 paired plasma samples collected at the same time from metastatic prostate patients showed discordant results. Complete congruence was shown in only 7.5% of cases, 15% had partial congruence and 40% had no congruence at all [32]. In addition, SNV at very low VAF should be taken into account cautiously, in accordance with the disease history, and when needed, confirmed by additional cfDNA analysis; tissue analysis when possible; or when applicable, the use of more appropriate liquids than plasma [33].

### 2.5. Copy Number Variations

Both FDA-approved panels have the capacity to detect amplifications for a subset of genes (310 out of 324 genes for F1LCDx and 18 out of 73 for G360). Only F1LCDx reports homozygous deletions, which is an important piece of information, especially when affecting tumor-suppressor genes. The possibility to use cfDNA for copy number profiling has been well-documented. Nevertheless, the CNV are only a qualitative parameter without indication of copy number (or ratio) and without distinction between real gene amplification or polysomy.

In neuroblastoma, the overall genomic profiles obtained using an OncoScan array (Affymetrix) on cfDNA were concordant in 97% (47/48) of the cases with the overall profile obtained using array comparative genomic hybridization (aCGH) on tissue samples [34].

The same excellent concordance was seen for the human epidermal growth factor receptor 2 (*HER2*) copy number comparing the results obtained with NGS performed on cfDNA with the G360 technology to fluorescent in situ hybridization (FISH) or IHC performed on matched tumor tissue. Indeed, for 75 metastatic colorectal cancer patients, the positive percent agreement between NGS and FISH/IHC was 82%, the negative percent agreement was 83% and the overall agreement reached 83%. Seven patients with an *HER2* amplification on tissue samples not confirmed on cfDNA had a significantly lower cfDNA fraction than the others, and six patients showed *HER2* amplification on cfDNA samples not identified on the tissue. This discordance can be explained by the fact that the tissue samples were collected before the initiation of anti-*EGFR* therapy, whereas cfDNA samples were obtained after disease progression [35].

### 2.6. Fusion/Rearrangement

Rearrangements and fusions can be detected by both FDA-approved panels at very low frequencies ranging from 0.2 to 0.9%. Positive percent agreement (PPA) between tissue and plasma kinase fusion detection is close to 70% (96 cfDNA confirmed fusions/137 tissue fusions) using F1LCDx. Tumor fraction influences the concordance of fusion detection as indicated by the median cfDNA fraction of 2.2% in the concordant situation versus 0.37% for the discordant situations (*p*-value < 0.001). With a cfDNA fraction of ≥1%, PPA reaches 85% [17].

Both FDA-approved panels use DNA as starting material for fusion detection. Although the DNA extracted from plasma samples is often of high quality, it presents several disadvantages. For example, the detection of gene rearrangements involving large intronic regions (more than 25 kb) can be challenging, especially using a capture-based approach [36]. It can be an issue in case of an unusual breakpoint or if the fusion partner has never been characterized before. In addition, another limitation is the discordance between DNA rearrangements identified and their RNA expression. In a study where 23 NTRK rearrangements were identified in a pan-cancer cohort, two were neither confirmed using a RNA-based NGS nor using pan-TRK IHC [37], and corresponded to the so-called bystander rearrangements.

For these reasons, the European Society for Medical Oncology (ESMO) recommends to use an RNA-based targeted approach for *NTRK1/2/3* [38] or *RET* fusion [39] detection to confirm predicted fusion transcripts.

A large study focused on 254 DNA-seq driver negative lung adenocarcinomas (no hotspot mutation, amplification or rearrangements) justifies these recommendations, showing that performing RNA-seq on these cases allowed for the identification of an actionable alteration (kinase fusion or *MET* exon 14 skipping) in 13% of cases [40].

## 3. Early Detection of Complex Drug-Resistance Mechanisms

Large NGS panels performed on cfDNA allow early detection of complex therapeutic resistance mechanisms.

For example, patients with advanced colorectal cancer without canonical mutations in the RAS/RAF pathway can be treated by a combination of chemotherapy plus antiepidermal growth factor receptor (*EGFR*) monoclonal antibody [41]. In some case reports, after 12 cycles of FOLFOX plus Panitumumab and maintenance therapy with 5-fluorouracil plus Panitumumab, several resistance mutations appeared. Many mutations identified using NGS were off-target resistance mutations localized in the mitogen-activated protein kinase (MAPK) pathway and affecting hotspot amino acid *KRAS* p.(Gly12Ala), *KRAS* p.(Gly12Cys), *KRAS* p.(Gly12Arg), *KRAS* p.(Gly12Val) or *NRAS* p.(Asn61Leu). On-target resistance mutations were also identified in the *EGFR* gene affecting noncanonical positions p.(Gly465Arg), *EGFR* p.(Ser464Leu), *EGFR* p.(Gly465Glu), *EGFR* p.(Val441Asp), *EGFR* p.(Val441Gly), *EGFR* p.(Lys489Glu), *EGFR* p.(Ile491Arg), *EGFR* p.(Ser464Lys), *EGFR* p.(Ser464Phe), *EGFR* p.(Ile491Lys). All mutations were identified at very low allelic frequency (ranging from 0.36% to 4.70%) [42].

Another example is the use of liquid biopsy to detect reversion mutations of breast-cancer susceptibility genes (*BRCA1 and BRCA2)* in high-grade ovarian carcinoma, in order to only treat patients who will benefit from poly(ADP) ribose polymerase (PARP) inhibitor. In a study performed on high-grade ovarian carcinoma, after treatment with a first-line platinum therapy, patients who developed BRCA reversion mutations had a significantly shorter progression-free survival under rucaparib compared to patients without reversion mutations (median 1.8 vs. 9.0 months; HR, 0.12; *p* < 0.0001) [43].

Moreover, liquid biopsy can be useful for the detection of intratumor heterogeneity, which has been described as a key point in the development of drug resistance [44]. In most cases, the resistance mechanism is not ruled by a single event but by a complex and multiple number of alterations. Liquid biopsy can be an interesting tool overcoming the sampling limitation of tissue biopsy and giving a complete representation of the subclonal architecture of resistance. In a cohort of 46 patients suffering from gastrointestinal cancer who developed an acquired resistance mechanism, NGS performed on liquid biopsy allowed detection of a molecular alteration in 76% of the cases (32/42 patients). In 53% of these cases, more than one mechanism was detected, reflecting the important tumor heterogeneity associated with acquired resistance (17/32 patients). Focusing on 23 patients of the cohort, both one solid and one liquid biopsy at time of progression, NGS performed on cfDNA was more sensitive than tissue NGS to detect resistance mechanisms. If an alteration was detected in 87% of the plasma samples (20/23 patients), only 48% of the tissue biopsy showed an alteration (11/23 patients), which in 91% of the cases was a single event (21/23 patients). In this cohort, only one resistance mutation was missed in the plasma sample due to the low allelic frequency of the mutation. Overall, in 78% of cases (18/23 patients), liquid biopsy revealed additional resistance mechanisms that would have not be detected with solid biopsy [45].

Finally, simultaneous detection of SNV, CNV and fusion in cfDNA when looking for resistance is crucial [46]. For example, cfDNA sequencing of 83 patients included in the AURA3 study using G360 demonstrate Osimertinib resistance mechanisms as heterogeneous as mutations in *EGFR*, *MET*, *HER2*, amplifications in *PIK3CA* or *FGFR3*, *RET* and *NTRK* oncogenic fusions [47]. Such cfDNA-based large panels are well-adapted to pleomorphic resistances.

## 4. Incidental Germline Variant Identification

The cfDNA-based assay also frequently leads to identification of germline alterations even if a small proportion of them are pathogenic and require genetic counseling. In fact, a mutation identified with an allelic frequency ranging from 40 to 60% must evoke a germline origin that in some cases will need to be explored through genetic testing and provide genetic counseling to the patient. In some pan-cancer studies using the G360 panel, a germline alteration is observed in 1.4% of the 10,888 unselected cfDNA samples and in most cases concerns *BRCA2*, *BRCA1* and *CDKN2A* [48].

Nevertheless, focusing only on VAF to identify the germline character of an alteration will lead to misclassification of many mutations identified in a context of elevated tumor fraction. Of 160 variants identified with an allelic frequency ranging from 40% to 60% in liquid biopsy with paired germline analysis, only 69% were confirmed to be germline. In this study, 96.3% of *BRCA2*, 90.9% of *BRCA1* and 86.7% of *CDH1* mutations were confirmed germline. Conversely, for genes with recurrent somatic mutations, 75% of *TP53* and 83.3% of *APC* mutations were somatic [49]. In our experience, we identified about 6% of cases with germline pathogenic variants detected by cfDNA based analysis, and half of them constitute incidental findings in a pan-cancer cohort of 801 patients. (Chabert A et al. submitted).

For these reasons, an alteration has to be interpreted considering the allelic frequencies of other mutations identified in the analysis, even by looking at the frequency of variants of unknown significance.

Another difficulty could be the identification of mosaic variants, such as some *TP53* or *BRCA2* variants with intermediate VAF (less than 40%) which could not be distinguished from somatic variants without additional analysis.

To solve incidental germline alterations, a solution could be to systematically associate a whole-blood NGS using the same panel and to subtract the unwanted alterations similar to what it is already done for whole exome/genome sequencing.

Of note, cfDNA NGS analysis performed on plasma cannot replace a whole-blood test for germline testing, as this NGS is not optimized for genetic testing (whole gene coverage and large rearrangement detection). A dedicated analysis on a whole-blood sample should be performed in case of germline variant suspicion, and certainly in the context of familial cancers. Finally, it is also important to recall that an orientation to genetic counseling should be limited to genes that already have established guidelines for the management of patients and family, such as the list provided by ESMO [50,51].

## 5. Occult Malignancy Identification

Between 2% and 17% of patients will be affected by multiple simultaneous primary cancers [52]. If in most cases the multiple malignancies are diagnosed based on clinical symptoms or as incidental findings on imaging, a small part of them can be discovered based on the analysis of cfDNA sequencing. Our team recently reported such cases [53]. The first case concerned a patient followed for lung cancer, for whom a *TMPRSS2*-*ERG* fusion was identified on cfDNA (a pathognomonic alteration for prostatic cancer). This patient then underwent a prostatic biopsy, confirming the diagnosis of prostate cancer. The second case was a patient treated for a cholangiocarcinoma. On the NGS performed on liquid biopsy, a *MYD88* p.(Leu265Pro) mutation was identified, which is a mutation present in 90% of Waldenström macroglonulinemia [54]. The hematologic diagnostic was confirmed on bone-marrow aspiration with the identification of mature B clones [53]. If in our experience, these two occult malignancies have been identified based on a fusion, and an SNV, targeted methylation of cfDNA will help us in the near future to know the localization of the tissue of origin [55].

## 6. Clonal Hematopoiesis

By combining large panels with lowering the LoD for the cfDNA-based NGS assays, mutations with VAF down to 0.1% are now detectable. It can be difficult to distinguish somatic mutations usually related to CH from the tumoral-related somatic mutation, all the more so that, for example, NSCLC patients harbor more clonal hematopoiesis variants than low-risk-of-cancer controls [56]. Prevalence of CH mutations in nonhematological malignancy has been reported at a frequency greater than 25% in a cohort of 8800 patients [57]. CH can make the interpretation of the clinical report complex.

As an example, although the CH origin of the mutation is of little doubt when it affects genes such as *DNMT3A*, *TET2*, *ASXL1* or *JAK2* [58], the situation is less clear for classical oncogenic drivers such as *KRAS* or common tumor suppressors such as *TP53.* Indeed, *KRAS* mutations usually found in pancreatic [59], colorectal [60] or lung cancer [61] are also frequently identified in cfDNA analysis. If most of the time the mutation is related to the oncogenic process, in some cases the classical hotspot mutations have been found in the matching peripheral blood cells, leading to a potential misdiagnosis of an occult malignancy [62].

Another limit is encountered with clinical trials focusing on PARP inhibitor in the context of homologous reparation deficiency. Some genes such as *ATM*, *BRCA1*, *BRCA2*, *BARD1*, *BRIP1*, *CDK12*, *CHEK1*, *CHEK2*, *FANCL*, *PALB2*, *RAD51B*, *RAD51C*, *RAD51D* or *RAD51L* have gained in interest, but cfDNA results should be analyzed carefully and interpreted with caution. For instance, in 10% of men with prostate cancer, patients had CH variants in several of the concerned genes, essentially *ATM* [63]. This may lead to potential errors in referring patients to clinical trials.

Finally, CH mutations can also erroneously lead to considering liquid biopsy results contributive, to not performing a second analysis, or to conclude that there are no drivers nor resistance mutations in the extracted cfDNA. This issue can be avoided when the initial driver is known and not found on ctDNA, which is the highest argument to consider cfDNA as not contributive.

## 7. Points of Attention

Regarding the added value and limitations previously mentioned, the first question that the oncologist should ask themselves before prescribing a liquid biopsy is the timing for blood collection: Is it the right time to prescribe this analysis? If this question may seem trivial, it is nonetheless crucial. It is likely that no alteration will be identified if the patient has a disease with a very low tumor burden or undergoing response under their actual treatment [64].

Another key point is the variability of cfDNA shedding between the different histological types and the different cancer stages. For example, cfDNA detectability has been reported as high in small-cell lung cancer, prostate, uterine and hepatocellular carcinoma (91.1%, 87.9%, 77.6% and 77.1%, respectively), but lower for thyroid and renal cancers (41.8% and 56.4%, respectively). cfDNA detection also depends on the stage of the disease [65]. In a study, 47% of patients with stage I disease had detectable cfDNA and this fraction increased in stage II, III and IV (55%, 69%, 82%, respectively) [66].

Nevertheless, in a same histological type, variability is also function of metastatic sites affected. Indeed, in a cohort of 517 patients with advanced oncogene-addicted NSCLC, cfDNA was detected in 52% of patients with isolated central nervous system progression (CNS); in 84% of patients with extra CNS progression only; and in 92% of patients with extra and CNS progression [33].

In an MTB context, the early detection of tumor variants by cfDNA can lead to proposing a treatment alternative after exhaustion of the standard treatment lines. In a recent study, 28% of the patients were advised to enter a clinical trial (49/173 patients) after MTB discussion using liquid biopsy, tissue biopsy or both. Nevertheless, in the same study liquid biopsy seemed to be more likely to detect all alteration types, whereas tumor biopsy does not (OR 13.6, 95% CI 5.5 to 43.2, *p* < 0.001) [67]. However, if the clinical trial inclusion capacity of results from liquid biopsy seems better, in the majority of the clinical trials conducted by pharmaceutical companies, the inclusion can be carried out only based on NGS results performed on tissue biopsy. In the future and with increasing experience, inclusion criteria are supposed to be more permissive for accepting NGS liquid biopsy results. Indeed, it has been described that NGS performed on cfDNA had the capacity to increase the trial enrollment rate with respect to tissue (9.5% vs. 4.1%, *p*-value < 0.0001) [12] in clinical trial accepting results performed on cfDNA.

## 8. Guidelines for Interpretation of cfDNA Clinical Reports

As interpretation of cfDNA clinical reports can be challenging, we recommend the following criteria:

- Tumor Fraction: this is the first parameter to look at for panels offering this information, keeping in mind the risk of overestimation if germline mutations have not been filtered out. If not calculated, results must be interpreted carefully and tumor content should be discussed. An estimation can also be given focusing on VAF of commonly identified “tracker” alterations in the histology, for example, *APC* mutations in sporadic colorectal cancer [68] or *TP53* mutations in high-grade serous ovarian cancer [69].
- MSI: As unexpected result can be observed in case of low tumor fraction; PCR [70] and IHC [71] are still gold standards and must be performed on tissue in case of doubt. The MMR status by NGS with cfDNA is only a good screening approach for positive results.
- bTMB: High risk of overestimation exists if germline or CH mutations are not filtered out. A context of multiclonal resistance can also be a factor of overestimation. We recommend elevating the cut-off to estimate which patients will benefit from immunotherapy to 16 mutations/Mb, to interpret it in the context of other variants and to confirm the result on tissue if needed.
- SNV: Mutations identified must be interpreted taking into account VAF and tumor fraction. The absence of any putative tumor variants should lead to consider the result as noncontributive, especially without the tumor fraction information. Furthermore, a SNV identified at very low VAF should be confirmed on tissue.
- CNV: If up to now no literature exists on the risk of overestimating the number of copies, in our experience, reported amplifications must be interpreted with caution and to be confirmed on tissue with FISH or IHC as in case of polysomic samples, the number of copies will be artifactually overestimated with a cancer gene panel.
- Fusion/Rearrangement: Currently, FDA-approved tests use DNA for fusion identification. It can represent an issue in case of large intronic regions and does not give information about expression. For these reasons, we recommend using an RNA-based technique as a confirmation tool if needed on tissue.
- Germline alterations: Not all the mutations identified with VAF between 40 and 60% are germline mutations. Results must be interpreted taking into account the tumor fraction and VAF of known alterations in the histology. Suspected germline alteration has to be confirmed on whole blood by a validated technique and a geneticist has to be involved in MTB to plan a genetic counseling if needed.
- Occult malignancies: In our experience, identification of other malignancies based on liquid biopsy results is a possible situation, based on the presence of recurrent drivers that are specific for certain tumor types. For this reason, we recommend including an oncologist with different specialty and a hematologist in the MTB. The exploration of a second cancer can be proposed to clinicians if the suspicion is high.
- CH: The hematologic malignant potential of identified mutations with high VAF has to be discussed with a hematologist. A hematologic consultation can be planned if this risk is high or if abnormalities are seen on the complete blood count. Furthermore, suspected CH mutations occurring in genes that allow the inclusion in clinical trials must be confirmed.

## 9. Conclusions

Liquid biopsy is a Swiss army knife in an MTB context by aggregating many tested parameters in a single analysis (SNV, CNV, fusion, TMB, microsatellite status). It allows for early detection of actionable variants and of resistance mechanisms, and reflects the tumor heterogeneity in a short turnaround time. This easy access to molecular information will probably ease the clinical trial inclusion and better help to manage patients at relapse and even preceding radiological relapse.

Nevertheless, the prescribing physician should handle liquid biopsy with care and be aware that he may face incidental findings such as germline alterations, clonal hematopoiesis mutations and occult malignancies that can be challenging. In these situations or in case of a negative liquid biopsy result, orthogonal testing of a tissue specimen should be considered if clinically indicated.

In the near future, cfRNA will likely be implemented in molecular analyses performed on liquid biopsies. It will provide a more comprehensive snapshot of the biological network and be a more accurate tool for fusion analyses [72].

For these reasons, with liquid biopsy the multidisciplinary aspect of solid-tumor MTB should be redefined. The involvement of new experts such as oncologists specializing in precision medicine, clinical hematologists, molecular biologists specializing in oncogenetics and hematology, molecular pathologists and geneticists become essential to ensure the optimal interpretation of molecular reports or adequate treatment tailoring.

## Figures and Tables

**Table 1:** Technological characteristics of the two FDA-approved cfDNA NGS. SNV: single-nucleotide variant, TMB: tumor mutational burden, LoD: limit of detection, G360: Guardant360^®^ CDx, F1LCDx: FoundationOne^®^ Liquid CDx.

Characteristics		G360	F1LCDx
Starting material	Whole blood	2 × 8.5 mL	2 × 10 mL
Alterations types	SNV	73 genes	311 genes
	Copy number variations	18 genes (amplification only)	310 genes
	Fusions/Rearrangements	8 genes	324 genes
	Microsatellite status	Yes	Yes
	TMB	No	Yes
Turnaround time	Announced	7 calendar days	Less than 2 weeks
LoD	SNV-indels 95–100%	0.20–0.25%	0.4–0.82%
	Fusions/Rearrangements	0.20%	0.37–0.90%
Tumor fraction		No	Yes
